# Navigating the Diagnostic Challenges in Lymph Node Cytology: The Case of Reactive Hyperplasia

**DOI:** 10.1111/cyt.13455

**Published:** 2024-11-05

**Authors:** Elena Vigliar, Gennaro Acanfora, Mauro Buono, Claudio Bellevicine, Marco Picardi, Giancarlo Troncone

**Affiliations:** ^1^ Department of Public Health University of Naples, “Federico II” Naples Italy; ^2^ Unit of Medical Physics and Radio Protection A.O.U Policlinico Federico II Naples Italy; ^3^ Department of Clinical Medicine and Surgery University of Naples, “Federic II” Naples Italy

## Abstract

Fine needle cytology (FNC) is a pivotal diagnostic tool for distinguishing between benign and malignant lymphadenopathies mainly because of its minimal invasiveness, cost‐effectiveness and accuracy. A major requirement for maximising diagnostic accuracy is proper sample management of aspirated cellular material. In this diagnostic process, the morphological evaluation of adequate smears is paramount, guiding cytopathologists in the selection of appropriate ancillary tests through the recognition of cell size and patterns of distribution. Here, we describe a peculiar ‘concentric ovals distribution pattern’, frequently observed in the FNC of benign reactive lymph nodes, which may represent an aid in the cytological diagnosis of reactive hyperplasia.

## Graphical abstract

The peculiar ‘concentric ovals distribution pattern’ frequently observed in the FNC of benign reactive lymph nodes may represent an aid in the cytological diagnosis of reactive hyperplasia.

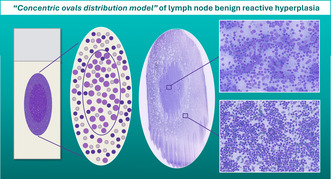

## 1 Introduction: The Role of FNC in Evaluating Lymphadenopathies

Being minimally invasive, cost effective and highly accurate, fine needle cytology (FNC) is commonly employed as a first‐line approach to diagnose lymphadenopathies [1]. Moreover, the possibility of combining ultrasound (US) and computerised tomography (CT) to FNC has enabled clinicians to reach and sample a higher number of lymph node. Recently, the Sydney System and, later, the WHO Reporting System for Lymph Node Cytopathology aimed to standardise the diagnostic procedures and reporting of lymph node FNC by providing key diagnostic cytopathological features, suggestions for standardised reports, and management recommendations [2, 3, 4], including clinical and/or imaging follow‐up, further ancillary testing and surgical excision. Thus, although the primary aim of lymph node cytology is to distinguish between benign and malignant processes, a second diagnostic level aimed at identifying more specific benign or malignant entities is recommended to tackle the diagnostic challenges associated with lymphadenopathies [2]. In this paper, we report the ability of FNC to detect a peculiar distribution pattern of benign lymph nodes with reactive hyperplasia.

## 2 Sample Management Is Crucial for Diagnostic Accuracy

Proper management of aspirated cellular material is pivotal to maximise diagnostic accuracy of lymph node FNC. Although haemodilution may occur in highly vascularised lymph nodes or in patients receiving antithrombotic medications, an adequately performed lymph node FNC often yields semisolid material [5]; therefore, a small drop of material should be placed near the frosted edge of the slide and a ‘one‐step’ smearing technique should be preferred [6]. At gross examination, the obtained smear will generally display an ovoid shape; after it is air‐dried and Romanovsky stained, the smear will turn intensely purple owing to the presence of abundant lymphoid cells.

## 3 Morphology Matters: The Pattern‐Based Approach

Generally, various ancillary techniques can be applied to lymph node FNC samples, such as immunocytochemistry, flow cytometry (FC) and molecular studies. Nonetheless, the morphological evaluation remains the first step of the diagnostic process, as it can also help cytopathologists choose the most appropriate ancillary test.

**FIGURE 1:**
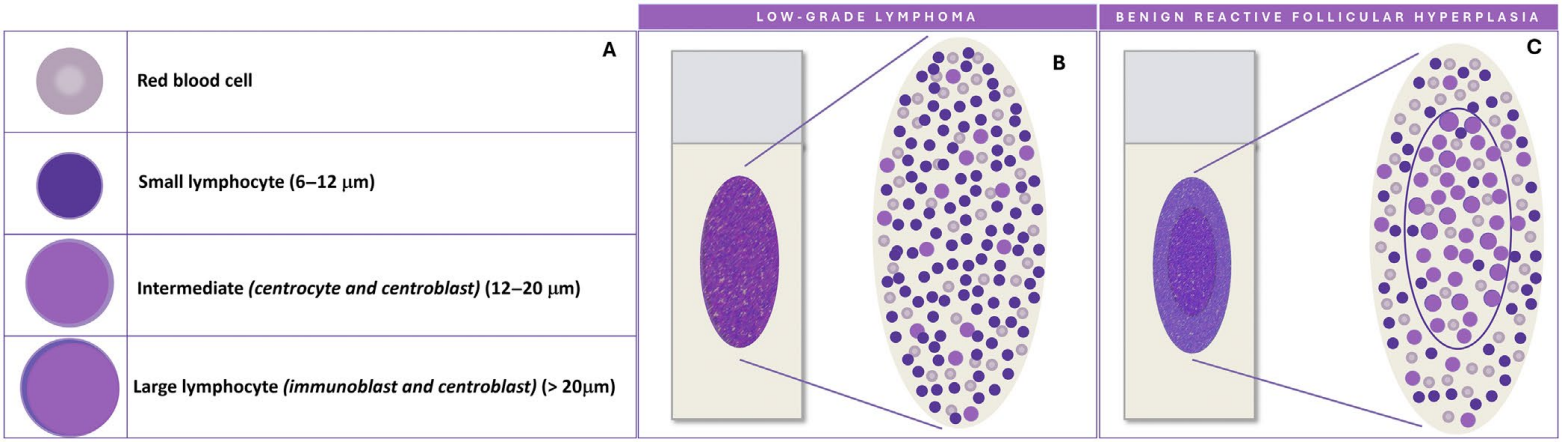
(A) Schematic representation of the sizes of lymphocytes. (B) Schematic representation of the monomorphic pattern of low‐grade lymphomas. (C) Schematic representation of the ‘concentric ovals distribution model’ observed in benign reactive follicular hyperplasia, in which intermediate and large cells tend to be more prominently represented in the middle of the slide, while smaller lymphocytes tend to be more numerous in the peripheral area.

Normal and reactive lymph nodes are both characterised by a varying proportion of lymphocytes in different maturation stages. Cell dimension varies considerably. Small lymphocytes measure 6–12 μm and include mature cells of primary follicles and mantle zone, and activated lymphocytes from the germinal centres. Large lymphocyte are > 20 μm and include immunoblasts and some centroblasts, whereas centrocytes and most centroblasts are intermediate in size, between small and large cells [5] (Figure 1A). Accordingly, an accurate smear microscopic evaluation is crucial to estimate the degree of variability in cell sizes and to identify the predominant cell populations. Indeed, polymorphous patterns, composed of small lymphocytes with a variable proportion of large lymphocytes, histiocytes and follicular dendritic cells (FDCs), are indicative of benign reactive lymph node hyperplasia; conversely, monotonous patterns, composed of small or large sized cells, as well as the presence of large pleomorphic cells, are suggestive of malignancy [2, 5].

## 4 Where Should the Pattern Assessment Be Conducted?

To improve consistency in cell size assessment, a some general rules should be followed: (1) the lymphocyte size should be determined by comparison with the size of red blood cells or nuclei of histiocytes; (2) cell size should be best determined in the thinner areas of the smear; (3) cell size evaluation should be avoided in cellular aggregates and in areas with air drying artefacts or damaged cells [5].

**FIGURE 2:**
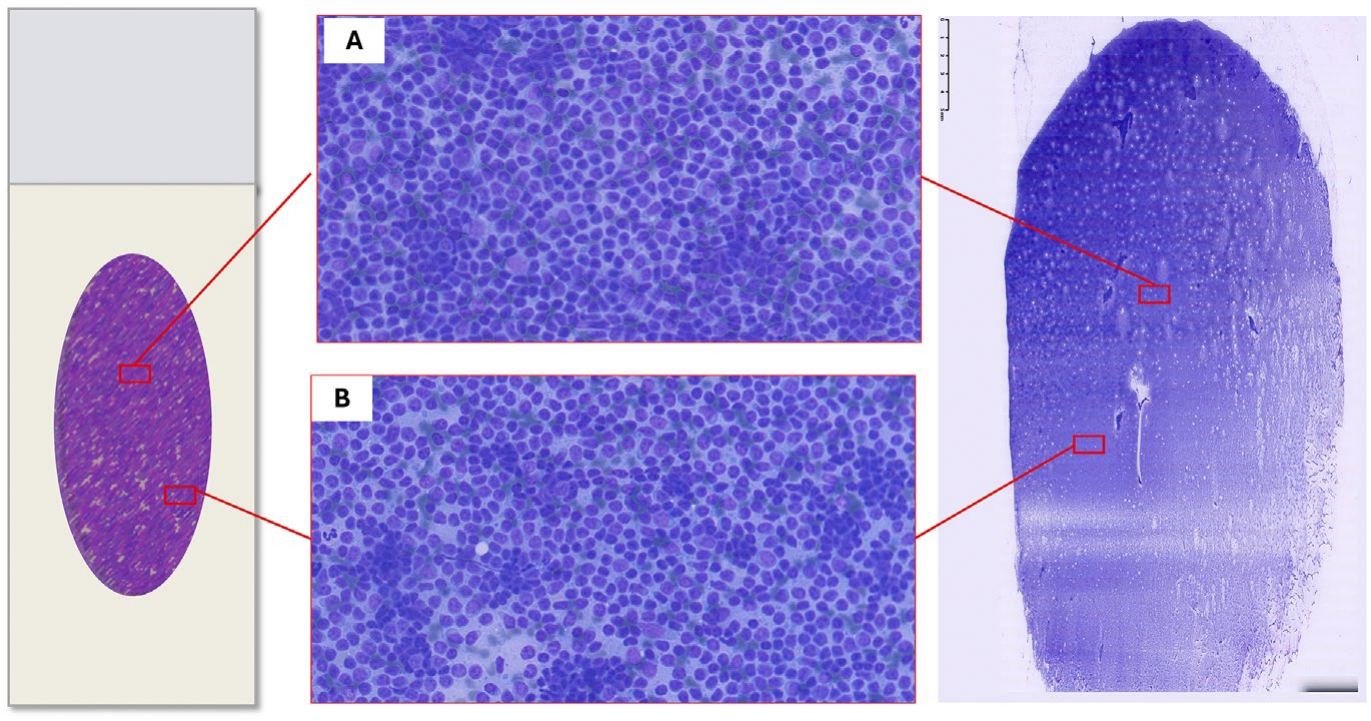
Fine needle cytology of low‐grade lymphoma confirmed by flow cytometry. The smear shows a monomorphic pattern featuring small lymphocytes equally distributed in the centre of the slide (A) and in the peripheral area (B). (Diff‐Quik stained smear, 20×).

**FIGURE 3:**
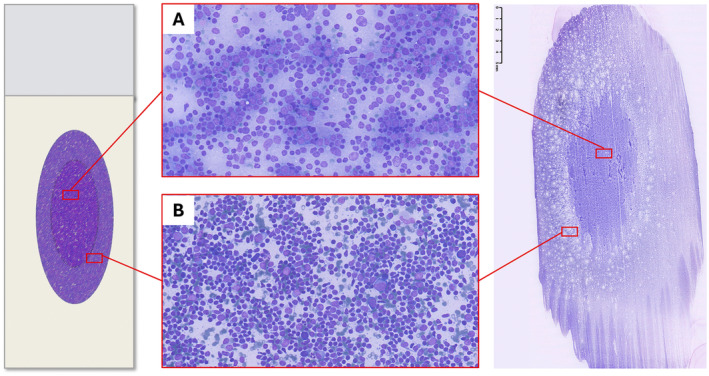
Fine needle cytology of lymph node benign reactive hyperplasia confirmed by flow cytometry. The smear shows the ‘concentric ovals appearance’, featuring intermediate and large lymphocytes in the middle of the slide (A) and small lymphocytes in the peripheral area (B). (Diff‐Quik stained smear, 20×).

**FIGURE 4:**
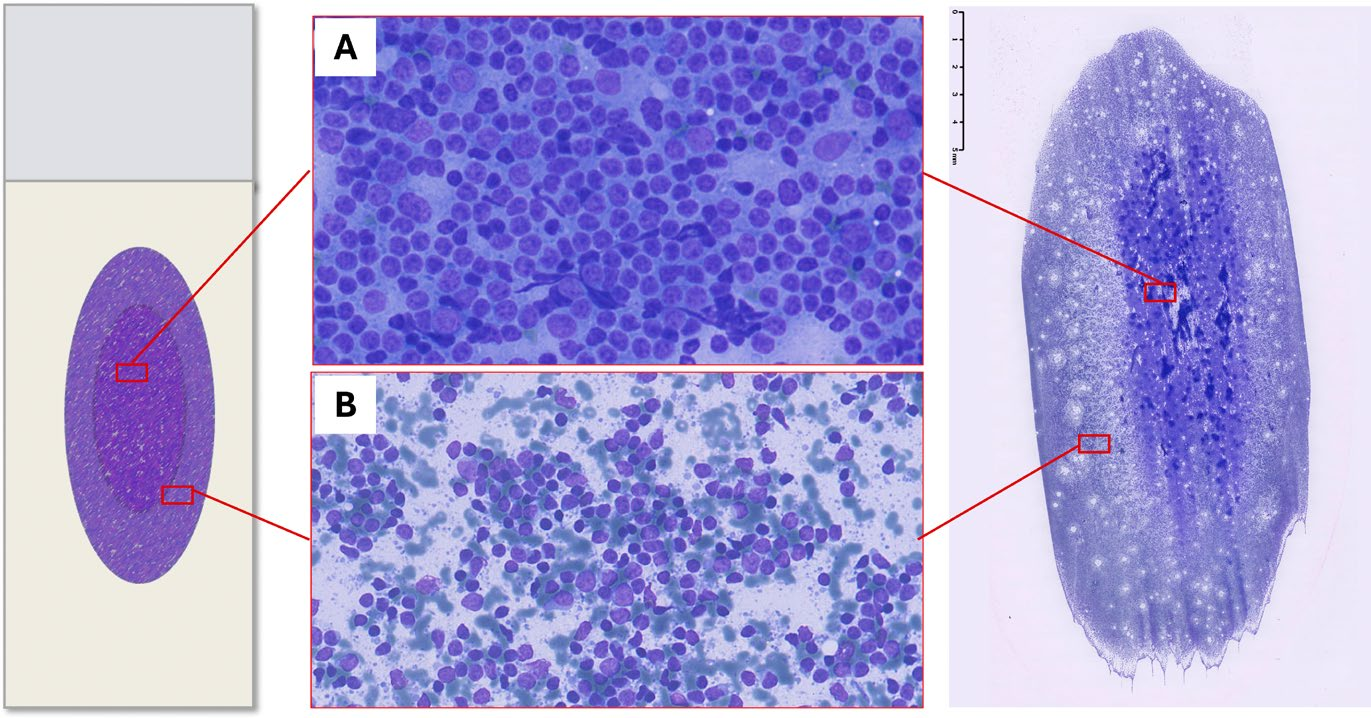
Fine needle cytology of lymph node benign reactive hyperplasia confirmed by flow cytometry. The smear shows the ‘concentric ovals appearance’, featuring intermediate and large lymphocytes in the centre of the slide (A) and small lymphocytes in the peripheral area (B). (Diff‐Quik stained smear, 20×).

Pattern recognition should be carried out through a thorough evaluation of the smear; however, the variable distribution of various sized cells among different slide areas could represent a pitfall. Generally, the monomorphic pattern of low‐grade lymphomas is evident throughout the slide (Figures 1B and 2). Conversely, in our everyday practice, we frequently observe a peculiar pattern of cell distribution in smears sampled from benign reactive follicular hyperplasia in which the diagnosis is also confirmed by FC. In fact, although the overall pattern can be considered polymorphous, owing to the presence of a mixture of small, intermediate and large lymphocytes, the larger ones tend to localise in the middle of the slide, whereas the smaller ones tend to predominate in the peripheral area. Visually, such peculiar cellular distribution forms a ‘concentric oval pattern’. Accordingly, when examining these slides, cytopathologists should bear in mind that the formation of these concentric oval patterns is due not only to a higher number of cells in the central area, but also to the compartmentalisation of different‐sized lymphocytes (Figure 1C). This is a very important aspect to consider because if the focus remained only on the central portion of the slide, the cellular distribution on the slide could be misidentified as a monomorphic pattern of medium‐large cells (Figures 3 and 4). This phenomenon is even more evident in reactive lesions as they yield a high proportion of centrocytes and centroblasts—a pitfall for false positive diagnoses [7]. This is especially true in clinical settings where FC immunophenotyping is unavailable or in case of little or no experience in lymph node cytopathology (i.e., young pathologists working in centres with a low volume of lymph node FNCs).

## 5 Physical Explanation: The Hypothesis

The physical phenomena generated in the one‐step smearing technique [6] could result in compartmentalisation of lymphoid cell populations, with the larger cells tending to gather in the centre, and the smaller ones in the peripheral areas of the glass slides.

**FIGURE 5:**
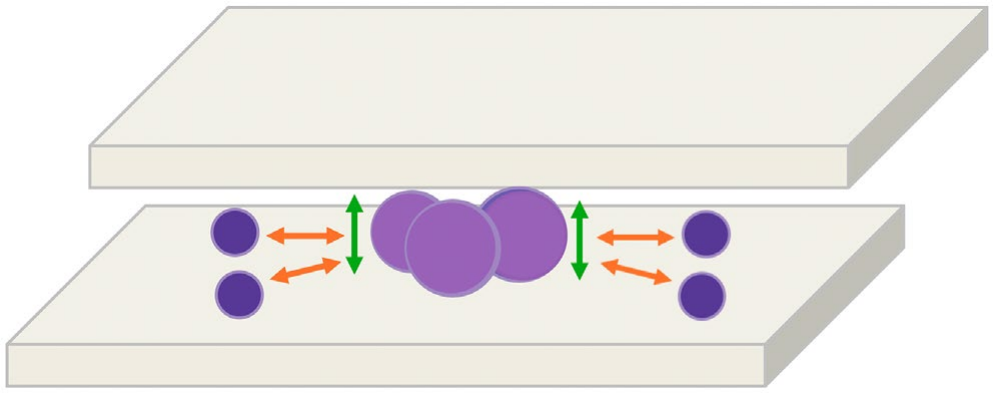
Schematic representation of cell distribution during smear: The cytological material is subjected to the pressure exerted by the two slides. Therefore, the larger cells will first come into contact with the overlapped glass slide (green arrows), tending to push some of the smaller cells from the centre towards the edges of the slide (orange arrows).

In fact, we speculate that such compartmentalisation is due to the fact that, when the smearing slide touches the stationary slide, cytological material moves because of the pressure played between the two slides. In particular, when the larger cells come into contact with the overlapping glass slide, they will remain in the centre of the slide, eventually pushing the smaller cells from the centre towards the edges (Figure 5).

Besides our physical hypothesis, we speculate that FDCs could also play a role in cell compartmentalisation. In fact, by forming a stable network and an intimate interaction with large germinal centre cells, FDCs might hamper the spreading of larger cells.

## 6 Conclusion

Recognising the potential pitfalls caused by variable cell distribution across glass slides is crucial to evaluating lymph node smear patterns. Specifically, in cases of benign reactive follicular hyperplasia, larger cells could be concentrated in the middle of a smear, which might misleadingly suggest a monomorphic population of medium to large cells. As this evidence is purely observational, a blind comparison of two different series of proven benign reactive hyperplasia and non‐Hodgkin lymphoma could confirm the described ‘concentric ovals distribution pattern’.

Lastly, we argue that a digital pathology approach coupled with digital analytics tools could demonstrate the consistency of this morphological distribution pattern of lymphocyte by evaluating the average cell size in different areas of the slide.

## Author Contributions

Conceptualisation: Elena Vigliar. Resources: Elena Vigliar and Claudio Bellevicine; writing – original draft preparation. Elena Vigliar, Gennaro Acanfora, Mauro Buono and Giancarlo Troncone. Writing – review and editing: all authors. All authors have read and agreed to the published version of the manuscript.

## Conflicts of Interest

Giancarlo Troncone received personal fees (as speakers bureau or advisor) from Roche, MSD, Pfizer and Bayer, for work unrelated to the current paper. The other authors declare no conflicts of interest.

## Data Availability

Data sharing is not applicable to this article as no datasets were generated or analysed during the current study.
